# Clinical Characteristics of Bloodstream Infection in Immunosuppressed Patients: A 5-Year Retrospective Cohort Study

**DOI:** 10.3389/fcimb.2022.796656

**Published:** 2022-04-04

**Authors:** Hongxia Lin, Lili Yang, Jie Fang, Yulian Gao, Haixing Zhu, Shengxiong Zhang, Hanssa Dwarka Summah, Guochao Shi, Jingyong Sun, Lei Ni, Yun Feng

**Affiliations:** ^1^ Department of Respiratory and Critical Care Medicine, Ruijin Hospital Affiliated Shanghai Jiao Tong University School of Medicine, Shanghai, China; ^2^ Institute of Respiratory Diseases, School of Medicine, Shanghai Jiaotong University, Shanghai, China; ^3^ Institute of Respiratory Diseases, People’s Hospital of Fuyang City, Fuyang, China; ^4^ Department of Pharmacy, Ruijin Hospital, School of Medicine, Shanghai Jiaotong University, Shanghai, China; ^5^ Department of Respiratory and Critical Care Medicine, Poudre D’Or Chest Hospital, Rivière du Rempart, Mauritius; ^6^ Department of Laboratory Medicine, Ruijin Hospital, Shanghai Jiao Tong University School of Medicine, Shanghai, China

**Keywords:** bloodstream infection, different subgroups, mortality, immunosuppressive states, prognosis

## Abstract

**Introduction:**

Immunosuppressed patients with bloodstream infection are at risk of mortality. Our objective was to assess the independent risk factors of bloodstream infection with mortality in immunosuppressive states.

**Methods:**

The medical data of a total of 896 patients who were hospitalized in our hospital were collected from January 2015 to December 2019. Evaluation of the independent risk factors of mortality was done by univariate and multivariate logistic regression analyses.

**Results:**

Of the 896 immunosuppressed patients with bloodstream infection, 698 had over 60-day survivals and 198 had 60-day mortality. In our study, PCT (mean ±; standard: 11.40 ±; 31.89 µg/l vs. 62.45 ±; 17.10 µg/l, p = 0.007) and presence of age >60 years (40% vs. 14.19%, p = 0.001) were significantly different between situations with and without 60-day survivals in both univariate and multivariate logistic regression analyses. Age >60 years and PCT could be used as indicators for bloodstream infection with 60-day death in immunosuppressive states; the OR (95% CI) were 1.532 (1.099–2.135) and 2.063 (1.413–3.013), respectively. In different subgroups, PCT and age were also independent risk factors of blood system diseases, *Klebsiella pneumoniae* infection, diabetes, and ICU-stay subgroups.

**Conclusions:**

Age and PCT were independently associated with mortality in immunosuppressive states, which may help to identify the highly risky situation of bloodstream infection in immunosuppressive states.

## 1 Introduction

Immunosuppressive (IS) status refers to low immune response. Generally, typical immunosuppressive status includes patients with hematological diseases, tumor, oral immunosuppressive medications, solid-organ transplant, stem cell transplant, or bone marrow transplant, and atypical immunosuppressive status includes patients with diabetes mellitus, liver cirrhosis, and burns (Mylotte and Tayara, 2000; Li et al., 2021; Willis et al., 2021). Studies revealed that immunosuppression could result from critical surgery as well (Si et al., 2021), and sometimes postoperative patients are susceptible to infections caused by opportunistic pathogens (Mada et al., 2020; Shipman et al., 2021). IS patients with infections may present with atypical signs and symptoms. Consequently, there is possibly a delay in diagnosis and treatment resulting in a poor prognosis of IS patients (Turkkan et al., 2021). IS status is closely related to mortality, and IS is associated with diverse pathogens including infection with bacteria, fungi, and viruses (McCann et al., 2004; Lindell et al., 2020; Li et al., 2021). Moreover, the clinical manifestations lack specificity. Thus, some indicators are needed to evaluate the clinical outcome. Previous investigations revealed that indicators like procalcitonin (PCT), age, intensive care unit (ICU) stay, malignancy, resistant bacterial infection, stroke, sex, diabetes mellitus, chronic renal failure, and previous use of antibiotics could indicate a poor prognosis of bloodstream infection (Zhang et al., 2019; Blanchard et al., 2020; Barlas et al., 2021; Chen et al., 2021; Jain et al., 2021; Zduniak et al., 2021).

IS status is a biologically feasible and widely acknowledged risk factor for mortality in patients with bloodstream infection, but the relationship between IS status and mortality in cases of bloodstream infection remains unclear. To our knowledge, there is no study assessing the significant predictors of mortality in bloodstream infection in patients with IS status.

To assess the clinical characteristics and the importance of risk factors related with mortality and to provide predictors of mortality in bloodstream infection associated with IS status, a retrospective analysis of all the cases of confirmed bloodstream infections in our hospital was conducted.

## 2 Materials and Methods

### 2.1 Subjects

The aims of this retrospective study were to evaluate the clinical characteristics in IS patients with organ tumor, hematological diseases, transplantation, autoimmune diseases on immunosuppressive therapy, diabetes, liver cirrhosis, postsurgery critical illness, and burns. We evaluated the prognostic risk factors not only in IS status but also in different IS subgroups.

Blood infections referred to various pathogenic microorganisms invading the blood, which could be confirmed by blood cultures. Coagulase-negative *Staphylococci* were considered real pathogens when isolated by multiple blood cultures. In our study, PCT was collected at the onset of infection. Body mass index (BMI) was recorded at hospitalization. In *Results*, the first values and corresponding percentages referred to the number and proportion (%) of different subgroups (primary diseases, etiologies, etc.) in the 60-day survival group. The second values and corresponding percentages referred to the number and proportion (%) of different subgroups (primary diseases, etiologies, etc.) in the 60-day death group. The precipitating factors referred to factors that caused bloodstream infections. The complications referred to the dysfunctions in other parts after bloodstream infection based on primary disease. The etiology resources with bloodstream infection referred to the primary sites of infection. Acute respiratory failure referred to the development of acute respiratory failure during the bacteremic event. Shock included septic shock, hypovolemic shock, and cardiogenic shock.

### 2.2 Measurements

To conduct this research, we compiled clinical data of 896 IS patients admitted in Ruijin Hospital from January 2015 to December 2019 who developed bloodstream infections during their hospital stay.

Patients meeting any of the following criteria for immunosuppression were selected: (1) tumor (organ and blood system); (2) solid-organ, stem cell, or bone marrow transplantation states; (3) autoimmune diseases with immunosuppressive therapy (more than 10 mg of prednisone or equivalent per day for at least 3 weeks or oral methotrexate, cyclosporine, azathioprine, or biological modifiers within 3 months); (4) poor diabetes control; (5) liver cirrhosis; (6) postoperative critical condition; and (7) burns (McCann et al., 2004; Blanchard et al., 2020; Lindell et al., 2020; Mada et al., 2020; Barlas et al., 2021; Turkkan et al., 2021).

### 2.3 Statistical Analysis

Patients were classified into 60-day mortality and 60-day survival, and data were analyzed according to different prognosis and different subgroups (underlying tumors, organ transplant, receiving long-term immunosuppressive treatment, diabetes mellitus, liver cirrhosis, postoperative critical condition, and burns). The clinical characteristics and prognostic factors of bloodstream infection for each of these groups were analyzed by using SPSS version 26.0. The proportions of IS-bloodstream infection in 60-day survival and 60-day mortality were compared by applying the χ^2^ test. The clinical factors associated with 60-day mortality in IS-bloodstream infection patients were evaluated by using univariate and multivariable logistic regression analyses. Body mass index (BMI), age, and PCT were analyzed as continuous variables. The χ^2^ test or Fisher exact test was used to access the categorical variables, and the Student t-test was applied to evaluate continuous variables. A two-sided p value <0.05 was statistically significantly.

### 2.4 Statement of Ethics Compliance

All analyses were based on previous clinical data and the study obtained ethical clearance from Ruijin Hospital Affiliated to Shanghai Jiaotong University School of Medicine Ethics Committee with approval of patient informed consent exemption.

## 3 Results

### 3.1 The General Characteristics of the Study Population

**Figure 1:**
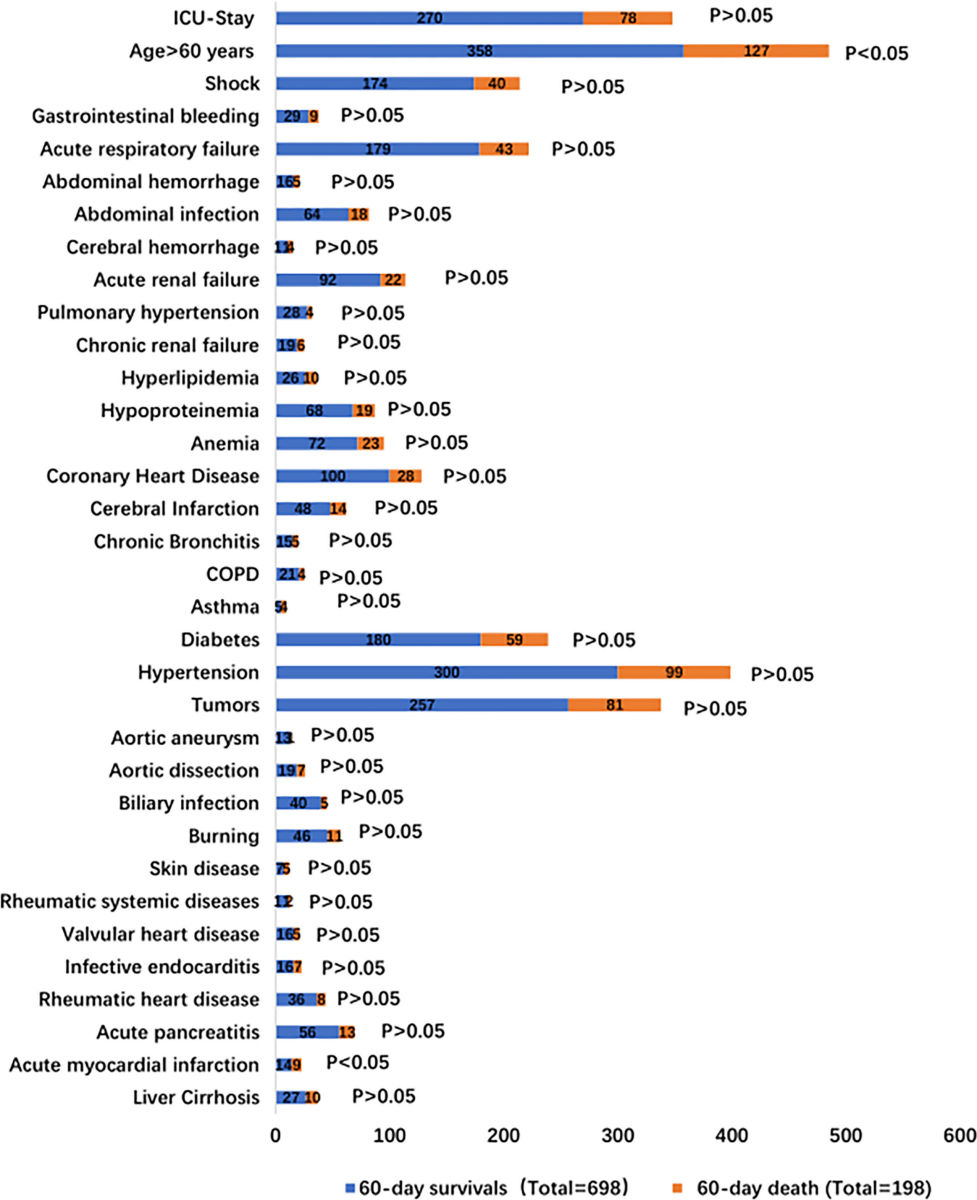
The clinical characteristics of bloodstream infection.

896 IS patients were selected for analysis of their clinical data in this study. They were categorized as 60-day survivors (698 patients) and of 60-day death (198 patients). The general characteristics are summarized in **Supplementary Table 1** and **Figure 1**. Approximately 71.21% (141/198) of the 60-day death patients were men with a mean ±; standard deviation age of 62.45 ±; 17.10 years, BMI of 23.54 ±; 3.97 kg/m^2^, and PCT of 20.49 ±; 43.59 µg/l; age and PCT were higher than those of 60-day survivors (p < 0.05). There was no difference in the sex, BMI, smoking, drinking, ICU stay, and underlying diseases between the two groups.

### 3.2 The Underlying Conditions With Bloodstream Infection Mortality in IS Status

The underlying conditions were liver cirrhosis (3.89% vs. 5.05%), acute myocardial infarction (2.01% vs. 4.54%), acute pancreatitis (8.02% vs. 6.57%), rheumatic heart disease (5.18% vs. 4.04%), infective endocarditis (2.29% vs. 3.53%), valvular heart disease (2.29% vs. 2.52%), rheumatic systemic diseases (1.58% vs. 1.01%), skin disease (1.00% vs. 2.52%), burns (6.59% vs. 5.56%), biliary infection (5.73% vs. 2.52%), aortic dissection (2.72% vs. 3.53%), aortic aneurysm (1.82% vs. 0.50%), and tumors (36.82% vs. 40.91%) (**Supplementary Table 2** and **Figure 1**). Patients with tumors included patients with different malignancies: hematological malignancies (34.24% vs. 35.80%), gastrointestinal cancer (52.92% vs. 51.85%), respiratory system tumors (3.89% vs. 4.94%), peritoneal malignant tumor (4.28% vs. 3.70%), and urinary system tumors (3.11% vs. 2.47%). There was no difference in the underlying disease between the two groups (**Supplementary Table 3**).

### 3.3 The Precipitating Factors, Complications, and Etiology Resources With Bloodstream Infection Mortality in IS Status

**Figure 2:**
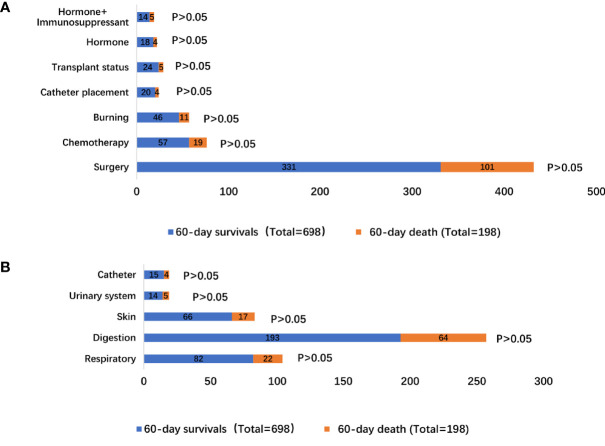
Distribution of bloodstream infection according to 60-day survivals and 60-day death stratifications: **(A)** different inducement of infection; **(B)** resources of infectious etiologies.

The precipitating factors for bloodstream infections in patients with IS status were mainly surgery (47.42% vs. 51.01%), chemotherapy (8.17% vs. 9.60%), and burns (6.59% vs. 5.56%). The complications were mainly acute renal failure (13.18% vs. 11.11%), abdominal infection (9.17% vs. 9.09%), acute respiratory failure (25.64% vs. 21.72%), and shock (24.93% vs. 20.20%). The sources of infection were mainly the respiratory tract (11.75% vs. 11.11%), digestive tract (27.65% vs. 32.32%), and skin (9.46% vs. 8.59%). There was no difference in the precipitating factors, complications, and sources of infection between the two groups (**Supplementary Table 4** and **Figures 1**, **2**).

### 3.4 The Etiology of Bloodstream Infections in IS Status

**Figure 3:**
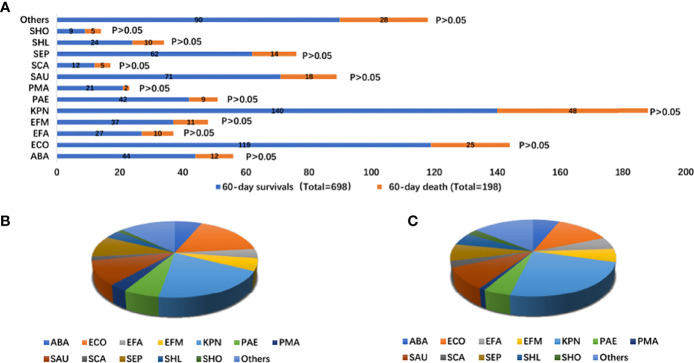
Distribution of infectious microorganisms of bloodstream infection according to 60-day survivals and 60-day death stratifications **(A)**; proportion of types of infectious microorganisms of bloodstream infection with 60-day survivals (total = 698) **(B)**; proportion of infectious microorganisms of bloodstream infection with 60-day death (total = 197) **(C)**. ABA, Acinetobacter baumannii; ECO, Escherichia Coli; EFA, Enterococcus faecalis; EFM, Enterococcus faecium; KPN, Klebsiella pneumoniae; PAE, seudomonas aeruginosa; PMA, Stenotrophomonas maltophilia; SAU, Staphylococcus aureus; SCA, Paratyphoid C; SEP, Staphylococcus epidermidis; SHL, Staphylococcus haemolyticus; SHO, Staphylococcus hominis.

The pathogens isolated in the patients were mainly *Klebsiella pneumoniae* (20.06% vs. 24.24%), *Escherichia coli* (17.05% vs. 12.53%), *Staphylococcus aureus* (10.17% vs. 9.09%), and *Staphylococcus epidermidis* (8.88% vs. 7.07%). There was no difference in the infectious microorganisms between the two groups (**Supplementary Table 4** and **Figure 3**).

### 3.5 Antibacterial Therapy in IS Status

The antibiotics were mainly third-generation cephalosporins (19.20% vs. 15.15%), carbapenems (60.74% vs. 71.72%), vancomycin (28.37% vs. 33.84%), linezolid (15.04% vs. 14.14%), and fluoroquinolones (13.18% vs. 9.60%). There was no difference in the antibacterial therapy between the two groups (**Supplementary Table 4** and **Supplementary Figure 1**).

### 3.5 Risk Factors Associated With 60-Day Mortality

The 60-day mortality was assessed by univariable logistic regression analysis, age >60 years (OR: 1.537, 95% CI: 1.102–2.145, p = 0.011) and PCT >0.5 µg/l (OR: 2.046, 95% CI: 1.400–2.991, p < 0.001) were considered as having significant differences. Using each significant item in the univariable logistic regression analysis, we assessed the risk factors associated with 60-day mortality by multivariable logistic regression analysis. Age >60 years (OR: 1.532, 95% CI: 1.099–2.135, p = 0.012) and PCT >0.5 µg/l (OR: 2.063, 95% CI: 1.413–3.013, p < 0.001) were considered as having significant differences. Therefore, age >60 years and PCT >0.5 µg/l were independent risk factors for 60-day mortality (**Table 1**).

**Table 1:** Univariate and multivariate analyses of factors related to over 60-day mortality in immunosuppressed patients.

Variable (total = 896)	Univariate analysis		Multivariate analysis	
	OR (95% CI)	*p* value	OR (95% CI)	*p* value
Age >60 years	1.537 (1.102–2.145)	0.011	1.532 (1.099–2.135)	0.012
PCT >0.5 µg/l	2.046 (1.400–2.991)	p < 0.001	2.063 (1.413–3.013)	p < 0.001
Acute myocardial infarction	2.271 (0.950–5.428)	0.065	Not Applicable	Not Applicable

### 3.6 Risk Factors Associated With 60-Day Mortality in Different Subgroups

#### 3.6.1 Hematological Disease Subgroup

The analysis results of univariable and multivariable logistic regression analyses showed that age >70 years (OR: 3.920, 95% CI: 1.490–10.312, p = 0.006), PCT >0.5 µg/l (OR: 3.608, 95% CI: 1.050–12.395, p = 0.042) and bacteria resistant to extended spectrum cephalosporins (OR: 4.597, 95% CI: 1.647–12.832, p = 0.004) were independent risk factors for 60-day mortality (**Table 2** and **Supplementary Figure 2**).

**Table 2:** Risk factors associated with 60-day mortality in different subgroups by univariate and multivariate analyses.

Hematological disease subgroup				
Variable (total = 117)	Univariate analysis		Multivariate analysis	
	OR (95% CI)	*p* value	OR (95% CI)	*p* value
Age >70 years	3.214 (1.342–7.700)	0.009	3.920 (1.490–10.312)	0.006
PCT >0.5 µg/l	3.750 (1.199–11.729)	0.023	3.608 (1.050–12.395)	0.042
Bacteria resistant to extended spectrum cephalosporins	3.731 (1.466–9.495)	0.006	4.597 (1.647–12.832)	0.004
**Shock subgroup**				
Variable (total = 214)	Univariate analysis		Multivariate analysis	
	OR (95% CI)	*p* value	OR (95% CI)	*p* value
Acute myocardial infarction	Not Applicable	Not Applicable	9.556 (1.686–54.168)	0.011
**Diabetes subgroup**				
**Variable (total = 239)**	**Univariate analysis**		**Multivariate analysis**	
	**OR (95% CI)**	*p* value	**OR (95% CI)**	***p* value**
Age >60 years	2.084 (1.114–3.900)	0.022	2.105 (1.089–4.068)	0.027
PCT >0.5 µg/l	2.233 (1.105–4.512)	0.025	2.082 (1.005–4.313)	0.048
Tumor	1.886 (1.019–3.490)	0.043	Not Applicable	0.069
SHL	1.063 (1.018–1.109)	0.005	5.578 (1.059–31.296)	0.043
ICU admission subgroup				
Variable (total = 248)	Univariate analysis		Multivariate analysis	
	OR (95% CI)	*p* value	OR (95% CI)	*p* value
Female	1.885 (1.063–3.342)	0.030	Not Applicable	0.055
Age >60 years	1.942 (1.145–3.291)	0.014	1.812 (1.043–3.148)	0.035
Acute myocardial infarction	7.243 (1.301–40.320)	0.024	8.007 (1.251–51.270)	0.028
PCT >0.5 µg/l	3.006 (1.578–5.727)	0.001	2.725 (1.402–5.296)	0.003
Diabetes	1.828 (1.051–3.178)	0.033	1.874 (1.043–3.366)	0.036
**ABA infection subgroup**				
Variable (total = 56)	Univariate analysis		Multivariate analysis	
	OR (95% CI)	*p* value	OR (95% CI)	*p* value
Burning	Not Applicable	Not Applicable	6.800 (1.690–27.356)	0.007
**KPN infection subgroup**				
Variable (total = 188)	Univariate analysis		Multivariate analysis	
	OR (95% CI)	*p* value	OR (95% CI)	*p* value
Female	2.596 (1.079–6.249)	0.033	Not Applicable	0.061
Age >60 years	3.055 (1.508–6.190)	0.002	2.542 (1.226–5.268)	0.012
PCT >0.5 µg/l	3.140 (1.366–7.214)	0.007	2.731 (1.161–6.427)	0.021
**ECO infection subgroup**				
Variable (total = 56)	Univariate analysis		Multivariate analysis	
	OR (95% CI)	*p* value	OR (95% CI)	*p* value
PCT >0.5 µg/l	Not Applicable	Not Applicable	4.958 (1.405–17.489)	0.013
**Bacteria resistant to extended spectrum cephalosporin subgroup**				
**Variable (total = 130)**	**Univariate analysis**		**Multivariate analysis**	
	**OR (95% CI)**	*p* value	**OR (95% CI)**	***p* value**
Chemotherapy	3.077 (1.036–9.139)	0.043	Not Applicable	0.946
Hematological diseases	3.874 (1.508–9.958)	0.005	4.072 (1.008–16.448)	0.049
Diabetes	2.827 (1.141–7.005)	0.025	2.879 (1.119–7.407)	0.028
**Acute respiratory failure subgroup**				
Variable (total = 222)	Univariate analysis		Multivariate analysis	
	OR (95% CI)	*p* value	OR (95% CI)	*p* value
Age >45 years	3.083 (1.042–9.120)	0.042	3.140 (1.039–9.4932)	0.043
Acute myocardial infarction	9.077 (1.605–51.324)	0.013	9.383 (1.589–55.392)	0.004
**Gram-positive bacterial infection with glycopeptide antibiotic treatment subgroup**				
**Variable (total = 130)**	**Univariate analysis**		**Multivariate analysis**	
	**OR (95% CI)**	*p* value	**OR (95% CI)**	***p* value**
Rheumatic diseases	8.806 (1.689–45.919)	0.010	7.735 (1.199–49.927)	0.032
Shock	8.276 (3.474–19.718)	<0.001	6.554 (2.495–17.212)	<0.001
Acute myocardial infarction	4.687 (1.059–20.739)	0.042	Not Applicable	0.070
Acute renal failure	4.550 (1.579–13.111)	0.005	Not Applicable	0.466
PCT >0.5 µg/l	2.531 (1.102–5.815)	0.029	Not Applicable	0.375
**Gram-negative bacterial infection with carbapenem antibiotic treatment subgroup**				
Variable (total = 313)	Univariate analysis		Multivariate analysis	
	OR (95% CI)	*p* value	OR (95% CI)	*p* value
PCT >0.5 µg/l	3.137 (1.365–7.211)	0.007	3.141 (1.273–7.749)	0.013
ICU-stay	2.071 (1.189–3.606)	0.010	Not Applicable	0.480
Burning	2.701 (1.056–6.912)	0.038	Not Applicable	0.696
Biliary infection	2.544 (1.137–5.696)	0.023	3.699 (1.454–9.409)	0.006
Shock	5.676 (3.128–10.298)	<0.001	4.671 (2.328–9.374)	<0.001
Acute renal failure	2.502 (1.212–5.165)	0.013	Not Applicable	0.296
Respiratory tract infection	2.194 (1.076–4.474)	0.031	Not Applicable	0.109
Skin infection	2.628 (1.083–6.374)	0.033	Not Applicable	0.470
**Extended spectrum cephalosporin-resistant Gram-negative bacterial infection subgroups**				
Variable (total = 124)	Univariate analysis		Multivariate analysis	
	OR (95% CI)	*p* value	OR (95% CI)	*p* value
KPN	2.565 (1.090–6.037)	0.031	Not Applicable	0.392
Drinking	4.286 (1.769–10.384)	0.001	4.273 (1.608–11.356)	0.004
Abdominal infection	4.720 (1.441–15.453)	0.010	Not Applicable	0.180
Shock	5.400 (2.145–13.594)	<0.001	3.701 (1.278–10.722)	0.016
Hypoproteinemia	3.750 (1.003–14.021)	0.049	Not Applicable	0.087

#### 3.6.2 Shock Subgroup

The independent risk factor in shock was acute myocardial infarction (OR: 9.556, 95% CI: 1.686–54.168, p = 0.011) (**Table 2** and **Supplementary Figure 2**).

#### 3.6.3 Diabetes Subgroup

In the diabetes subgroup, the independent risk factors with 60-day mortality were age >60 years (OR: 2.105, 95% CI: 1.089–4.068, p = 0.027), PCT >0.5 µg/l (OR: 2.082, 95% CI: 1.005–4.313, p = 0.048, and *Staphylococcus haemolyticus* (SHL) infection (OR: 5.578, 95% CI: 1.059–31.296, p = 0.043) (**Table 2** and **Supplementary Figure 2**).

#### 3.6.4 ICU Admission Subgroup

In the ICU-stay subgroup, the independent risk factors with 60-day mortality were age >60 years (OR: 1.812, 95% CI: 1.043–3.148, p value = 0.035), acute myocardial infarction (OR: 8.007, 95% CI: 1.251–51.270, p = 0.028), PCT >0.5 µg/l (OR: 2.725, 95% CI: 1.402–5.296, p = 0.003), and diabetes (OR: 1.874, 95% CI: 1.043–3.366, p = 0.036) (**Table 2** and **Supplementary Figure 2**).

#### 3.6.5 The Assessment of Mortality Risk According to Etiologies

The independent risk factors in cases with ABA infection were burns (OR: 6.800, 95% CI: 1.690–27.356, p = 0.007) (**Table 2** and **Supplementary Figure 2**).

The independent risk factors in cases with ECO infection were PCT >0.5 µg/l (OR: 4.958, 95% CI: 1.405–17.489, p = 0.013) (**Table 2** and **Supplementary Figure 2**).

The independent risk factors in KPN infection associated with 60-day mortality were age >60 years (OR: 2.542, 95% CI: 1.226–5.268, p = 0.012) and PCT >0.5 µg/l (OR: 2.731, 95% CI: 1.161–6.427, p = 0.021) (**Table 2** and **Supplementary Figure 2**).

In the extended-spectrum cephalosporin-resistant bacteria subgroup, the independent risk factors with 60-day mortality were hematological diseases (OR: 4.072, 95% CI: 1.008–16.448, p = 0.049) and diabetes (OR: 2.879, 95% CI: 1.119–7.407, p = 0.028) (**Table 2** and **Supplementary Figure 2**).

#### 3.6.6 Acute Respiratory Failure Subgroup

In the acute respiratory failure subgroup, the independent risk factors with 60-day mortality were age >45 years (OR: 3.140, 95% CI: 1.039–9.493, p = 0.043) and acute myocardial infarction (OR: 9.383, 95% CI: 1.589–55.392, p = 0.004) (**Table 2** and **Supplementary Figure 2**).

#### 3.6.7 Gram-Negative Bacterial Infection With Carbapenem Antibiotic Treatment, Gram-Positive Bacterial Infection With Glycopeptide Antibiotic Treatment, Extended Spectrum Cephalosporin-Resistant Gram-Negative Bacterial Infection Subgroup

In the gram-positive bacterial infection with glycopeptide antibiotic treatment subgroup, the independent risk factors with 60-day mortality were rheumatic diseases (OR: 7.735, 95% CI: 1.199–49.927, p = 0.032) and shock (OR: 6.554, 95% CI: 2.495–17.212, p < 0.001) (**Table 2** and **Supplementary Figure 2**).

In the gram-negative bacterial infection with carbapenem antibiotic treatment subgroup, the independent risk factors with 60-day mortality were PCT >0.5 µg/l (OR: 3.141, 95% CI: 1.273–7.749, p = 0.013), biliary infection (OR: 3.699, 95% CI: 1.454–9.409, p = 0.006), shock (OR: 4.671, 95% CI: 2.328–9.374, <0.001) (**Table 2** and **Supplementary Figure 2**).

In the extended-spectrum cephalosporin-resistant gram-negative bacterial infection subgroup, the independent risk factors with 60-day mortality were drinking (OR: 4.273, 95% CI: 1.608–11.356, p = 0.004) and shock (OR: 3.701, 95% CI: 1.278–10.722, p = 0.016) (**Table 2** and **Supplementary Figure 2**).

## 4 Discussion

To our knowledge, many studies assessed risk factors for different microbial infections in the immunocompromised and specific microbial infection in different types of IS. However, studies investigating overall IS status associated with bloodstream infection are scarce. Our study explored the clinical characteristics in IS patients with organ tumor, hematological diseases, transplantation, autoimmune diseases on immunosuppressive therapy, diabetes, liver cirrhosis, postsurgery critical illness, and burns. We evaluated the prognostic risk factors not only in IS status but also in different IS subgroups. Age and PCT were independent prognostic risk factors for IS patients. In the analysis of 896 IS patients, we also evaluated the prognostic risks of different IS subgroups, especially for hematological diseases, diabetes, and ICU admission.

Bloodstream infection was a severe complication of hematological diseases, especially in the occurrence of resistant bacteria (Castagnola et al., 2021). Previous investigations also showed that age and PCT were associated with poor outcome in hematological diseases (Mylotte and Tayara, 2000; Arora and Sahni, 2018; Stoma et al., 2019; Chen et al., 2021; Levy et al., 2021). The results were consistent with our research results. In patients with hematological diseases, the independent risk factors were age >70 years, PCT >0.5 µg/l, and bacteria resistant to extended-spectrum cephalosporins.

Some studies found that hypoglycemia presented a higher mortality in patients with diabetes, and patients with uncontrolled diabetes and bloodstream infection had a poor prognosis (Blanchard et al., 2020). Age, PCT, and microbial infection were associated with a poor prognosis in patients with diabetes (Kanafani et al., 2009; Arora and Sahni, 2018). Our study had similar findings. In the analysis of the diabetes subgroup, the prognostic risk factors were age >60 years, PCT >0.5 µg/l, and SHL infection.

Bloodstream infection is associated with a high mortality in ICU (Lindell et al., 2020). IS patients easily suffered from multiple infections resulting in admission to ICU. Infectious complications contributed to the highly mortality in IS status (Barlas et al., 2021). In our study, the conclusion was the same as in previous studies: age, PCT, and diabetes could be independent factors for ICU admission (Fraunberger et al., 2006; Martin et al., 2006; Blanchard et al., 2020). Moreover, rare investigations analyzed the relationship between bloodstream infection and acute myocardial infarction. The current research mainly evaluated the outcome of acute myocardial injuries in COVID-19 (Fardman et al., 2021; Kite et al., 2021). Our study presented that acute myocardial infarction could be a prognostic predicator of ICU admission in IS status.

Previous investigations showed that the clinical prognosis had a close relationship with the patient’s immune status (McCann et al., 2004; Lindell et al., 2020). Many studies presented that different immunosuppressive status had different independent risk factors for prognosis. For example, in hematopoietic stem cell transplant patients with ICU stay, the independent risk factors were age, underlying diseases, ICU admission time, degree of severity of organ failure, several level of critical diseases, and primary disease (Castagnola et al., 2021). In the hematological malignancies’ patients with ABA infection, the independent risk factors were Sequential Organ Failure Assessment (SOFA) score and antibiotic therapy (Zduniak et al., 2021). In the solid-organ transplant recipients, the independent risk factors were infected bacteria resistant to extended-spectrum cephalosporins, age, and antifungal drugs (Anesi et al., 2021). In the patients with cancer, the independent risk factors were comorbidities, age, underlying diseases (mainly hematological malignancies), infection resources, types of microbial infections, hypoalbuminemia, antibiotic therapy, antibiotic-resistant organisms, shock, and ICU admission (Gudiol et al., 2011; Marín et al., 2015; Torres et al., 2015; Satlin et al., 2016; Marín et al., 2019; Martinez-Nadal et al., 2020; Gudiol et al., 2021). Many studies mainly showed the prognostic risk factors of an immunosuppressive disease in different types of microbial infections or a microbial infection in different types of IS; the prognostic predictors of mortality in overall bloodstream infection in IS status were unclear. In our study, we analyzed the prognostic factors in overall IS patients and different IS status subgroups; age >60 and PCT >0.5 µg/l had a closely relationship with 60-day mortality in IS status.

This study demonstrated a close association between PCT and poor prognosis in IS patients. PCT is an acute reaction protein; previous studies showed that it is closely related with the severity of acute infection (Jacobs et al., 2018). Several investigations presented that PCT could be an indicator in blood system diseases, tumor, oral immunosuppressive medications, solid-organ transplant, stem cell transplant or bone marrow transplant, and diabetes (Mylotte and Tayara, 2000; Kanafani et al., 2009; Arora and Sahni, 2018; Jacobs et al., 2018; Stoma et al., 2019; Chen et al., 2021). The conclusions were similar with our study in which PCT presented a poor prognostic predictor. In our study, PCT >0.5 µg/l could be an independent risk factor for 60-day mortality in IS patients (OR: 2.063, 95% CI: 1.413–3.013, p < 0.001). In different subgroups, PCT >0.5 µg/l was the independent risk factor for blood system diseases (OR: 3.608, 95% CI: 1.050–12.395, p = 0.042), ECO infection (OR: 4.958, 95% CI: 1.405–17.489, p = 0.013), KPN infection (OR: 2.731, 95% CI: 1.161–6.427, p = 0.021), diabetes (OR: 2.082, 95% CI: 1.005–4.313, p = 0.048), ICU hospitalization (OR: 2.725, 95% CI: 1.402–5.296, p = 0.003), and gram-negative bacterial infection with carbapenem antibiotic treatment (OR: 3.141, 95% CI: 1.273–7.749, p = 0.013).

The study also demonstrated that age suggested a poor prognosis in IS patients. Because of the decrease in immune system function with age, elderly people were more prone to infections (Zhang et al., 2019). Previous studies showed that age could be an indicator in severe infection (Martin et al., 2006). Our study showed identical results with a high mortality in the elderly. In our study, age >60 years could be an independent risk factor for 60-day mortality in IS patients (OR:1.532, 95% CI: 1.099–2.135, p = 0.012). In the analysis of different subgroups, age >60 years as a significant prognostic factor of KPN infection (OR: 2.542, 95% CI: 1.226–5.268, p = 0.012), diabetes (OR: 2.105, 95% CI: 1.089–4.068, p = 0.027), and ICU hospitalization (OR: 1.812, 95% CI: 1.043–3.148, p = 0.035) were evaluated.

Several limitations should be taken into consideration. Firstly, the clinical data were collected in a single center, the final results may not represent other different medical institutions. Secondly, the composition of primary illness was mainly hematological diseases and gastrointestinal cancer, it may produce data bias. Thirdly, some IS subgroups included a small number of patients, large-scale research may be needed in the future.

## 5 Conclusions

The clinical characteristics of underlying diseases and etiological features had a rare difference between 60-day mortality and 60-day survival. Age >60 years and PCT >0.5 µg/l as significant indicators for death risk of bloodstream infection in immunosuppressive status. In the analysis of IS subgroups, the prognostic risk factors of diabetes were age >60 years, PCT >0.5 µg/l, and SHL infection, and those of ICU admission were age >60 years, acute myocardial infarction, PCT >0.5 µg/l, and diabetes.

## Data Availability Statement

The raw data supporting the conclusions of this article will be made available by the authors, without undue reservation.

## Ethics Statement

The studies involving human participants were reviewed and approved by the Ruijin Hospital Affiliated to Shanghai Jiaotong University School of Medicine Ethics committee. Written informed consent for participation was not required for this study in accordance with the national legislation and the institutional requirements.

## Author Contributions

Conception and design of the work: YF and LN. Data interpretation: GS, JS. Collecting data: HL, LY, JF, YG, HZ, SZ. Data analysis: HL, LY, JF, JS. Drafting the work or revising it critically for important intellectual content: HL, HS, YF. All authors contributed to the article and approved the submitted version.

## Funding

The present study was supported by the National Natural Science Foundation of China (No. 8217010254); (No. 82170086), Shanghai Shenkang Hospital Development Center Clinical Science and Technology Innovation Project (SHDC12018102), Shanghai Municipal Key Clinical Specialty (shslczdzk02202), and Shanghai Key Laboratory of Emergency Prevention, Diagnosis and Treatment of Respiratory Infectious Diseases (20dz2261100).

## Conflict of Interest

The authors declare that the research was conducted in the absence of any commercial or financial relationships that could be construed as a potential conflict of interest.

## Publisher’s Note

All claims expressed in this article are solely those of the authors and do not necessarily represent those of their affiliated organizations, or those of the publisher, the editors and the reviewers. Any product that may be evaluated in this article, or claim that may be made by its manufacturer, is not guaranteed or endorsed by the publisher.

## Supplementary Material

Supplementary Figure 1 Distribution of bloodstream infection according to 60-day survivals and 60-day death stratifications: different antibacterial therapy.

Supplementary Figure 2 Risk factors associated with 60-day mortality in different subgroups.

## References

[B1] Anesi J. A. Lautenbach E. Tamma P. D. Thom K. A. Blumberg E. A. Alby K.. (2021). Risk Factors for Extended-Spectrum β-Lactamase-Producing Enterobacterales Bloodstream Infection Among Solid-Organ Transplant Recipients. Clin. Infect. Dis. 72 (6), 953–960. doi: 10.1093/cid/ciaa190 32149327 PMC7958726

[B2] Arora R. Sahni N. (2018). Can Serum Procalcitonin Aid in the Diagnosis of Blood Stream Infection in Patients on Immunosuppressive Medications? Clin. Chim. Acta 483, 204–208. doi: 10.1016/j.cca.2018.05.002 29730396

[B3] Barlas T. İnci K. Aygencel G. Türkoğlu M. Tunçcan Ö. G. Can F.. (2021). Infections in Hematopoietic Stem Cell Transplant Patients Admitted to Hematology Intensive Care Unit: A Single-Center Study. Hematology 26 (1), 328–339. doi: 10.1080/16078454.2021 33818297

[B4] Blanchard F. Charbit J. van der Meersch G. Popoff B. Picod A. Cohen R.. (2020). Early Sepsis Markers in Patients Admitted to Intensive Care Unit With Moderate-to-Severe Diabetic Ketoacidosis. Ann. Intensive Care 10 (1), 58. doi: 10.1186/s13613-020-00676-6 32430795 PMC7237630

[B5] Castagnola E. Bagnasco F. Mesini A. Agyeman P. K. A. Ammann R. A. Carlesse F.. (2021). Antibiotic Resistant Bloodstream Infections in Pediatric Patients Receiving Chemotherapy or Hematopoietic Stem Cell Transplant: Factors Associated With Development of Resistance, Intensive Care Admission and Mortality. Antibiot. (Basel). 10 (3), 266. doi: 10.3390/antibiotics10030266 PMC8000765 33807654

[B6] Chen L. Han X. Li Y. Li M. (2021). Assessment of Mortality-Related Risk Factors and Effective Antimicrobial Regimens for Treatment of Bloodstream Infections Caused by Carbapenem-Resistant Enterobacterales. Antimicrob. Agents Chemother. 65 (9), e0069821. doi: 10.1128/AAC.00698-21 34228539 PMC8370219

[B7] Chen J. Huang J. Wang T. Xie C. (2021). Analysis of the Relationship Between Serum Amyloid Protein A, Procalcitonin, C-Reactive Protein, and Peripherally Inserted Central Catheter Infection in Patients With Malignant Tumor. Ann. Palliat. Med. 10 (5), 5359–5365. doi: 10.21037/apm-21-796 34107707

[B8] Fardman A. Zahger D. Orvin K. Oren D. Kofman N. Mohsen J.. (2021). Acute Myocardial Infarction in the Covid-19 Era: Incidence, Clinical Characteristics and in-Hospital Outcomes-A Multicenter Registry. PloS One 16 (6), e0253524. doi: 10.1371/journal.pone.0253524 34143840 PMC8213163

[B9] Fraunberger P. Wang Y. Holler E. Parhofer K. G. Nagel D. Walli A. K.. (2006). Prognostic Value of Interleukin 6, Procalcitonin, and C-Reactive Protein Levels in Intensive Care Unit Patients During First Increase of Fever. Shock 26 (1), 10–12. doi: 10.1097/01.shk.0000215319.06866.bd 16783191

[B10] Gudiol C. Albasanz-Puig A. Cuervo G. Carratalà J. (2021). Understanding and Managing Sepsis in Patients With Cancer in the Era of Antimicrobial Resistance. Front. Med. (Lausanne). 8, 636547. doi: 10.3389/fmed.2021.636547 33869250 PMC8044357

[B11] Gudiol C. Tubau F. Calatayud L. Garcia-Vidal C. Cisnal M. Sánchez-Ortega I.. (2011). Bacteraemia Due to Multidrug-Resistant Gram-Negative Bacilli in Cancer Patients: Risk Factors, Antibiotic Therapy and Outcomes. J. Antimicrob. Chemother. 66 (3), 657–663. doi: 10.1093/jac/dkq494 21193475

[B12] Jacobs L. Berrens Z. Stenson E. K. Zackoff M. Danziger-Isakov L. Lahni P.. (2018). Interleukin-27 as a Candidate Diagnostic Biomarker for Bacterial Infection in Immunocompromised Pediatric Patients. PloS One 13 (11), e0207620. doi: 10.1371/journal.pone.0207620 30475852 PMC6261028

[B13] Jain P. Galiya A. Luke Philip S. Mateti U. V. Supriya P. S. Gudi S. K.. (2021). Bacteriological Profile and Antimicrobial Resistance Pattern Among Patients With Sepsis: A Retrospective Cohort Study. Int. J. Clin. Pract. 75 (10), e14701. doi: 10.1111/ijcp.14701 34351692

[B14] Kanafani Z. A. Kourany W. M. Fowler V. G. Jr Levine D. P. Vigliani G. A. Campion M.. (2009). Clinical Characteristics and Outcomes of Diabetic Patients With Staphylococcus Aureus Bacteremia and Endocarditis. Eur. J. Clin. Microbiol. Infect. Dis. 28 (12), 1477–1482. doi: 10.1007/s10096-009-0808-3 19730900

[B15] Kite T. A. Ludman P. F. Gale C. P. Wu J. Caixeta A. Mansourati J.. (2021). International Prospective Registry of Acute Coronary Syndromes in Patients With COVID-19. J. Am. Coll. Cardiol. 77 (20), 2466–2476. doi: 10.1016/j.jacc.2021.03.309 34016259 PMC8128002

[B16] Levy I. Lavi A. Zimran E. Grisariu S. Aumann S. Itchaki G.. (2021). COVID-19 Among Patients With Hematological Malignancies: A National Israeli Retrospective Analysis With Special Emphasis on Treatment and Outcome. Leuk. Lymphoma. 18, 1–10. doi: 10.1080/10428194.2021.1966782 34405767

[B17] Li L. Hsu S. H. Wang C. Li B. Sun L. Shi J.. (2021). Characteristics of Viral Pneumonia in Non-HIV Immunocompromised and Immunocompetent Patients: A Retrospective Cohort Study. BMC Infect. Dis. 21 (1), 767. doi: 10.1186/s12879-021-06437-5 34362320 PMC8343364

[B18] Lindell R. B. Nishisaki A. Weiss S. L. Traynor D. M. Fitzgerald J. C. (2020). Risk of Mortality in Immunocompromised Children With Severe Sepsis and Septic Shock. Crit. Care Med. 48 (7), 1026–1033. doi: 10.1097/CCM.0000000000004329 32301846 PMC7311286

[B19] Mada P. K. Saldaña Koppel D. A. Al Shaarani M. Joel Chandranesan A. S. (2020). Primary Cutaneous Aspergillus Fumigatus Infection in Immunocompetent Host. BMJ Case Rep. 13 (2), e233020. doi: 10.1136/bcr-2019-233020 PMC7046428 32060111

[B20] Marín M. Gudiol C. Ardanuy C. Garcia-Vidal C. Jimenez L. Domingo-Domenech E.. (2015). Factors Influencing Mortality in Neutropenic Patients With Haematologic Malignancies or Solid Tumours With Bloodstream Infection. Clin. Microbiol. Infect. 21 (6), 583–590. doi: 10.1016/j.cmi.2015.01.029 25680311

[B21] Marín M. Gudiol C. Castet F. Oliva M. Peiró I. Royo-Cebrecos C.. (2019). Bloodstream Infection in Patients With Head and Neck Cancer: A Major Challenge in the Cetuximab Era. Clin. Transl. Oncol. 21 (2), 187–196. doi: 10.1007/s12094-018-1905-5 29948973

[B22] Martinez-Nadal G. Puerta-Alcalde P. Gudiol C. Cardozo C. Albasanz-Puig A. Marco F.. (2020). Inappropriate Empirical Antibiotic Treatment in High-Risk Neutropenic Patients With Bacteremia in the Era of Multidrug Resistance. Clin. Infect. Dis. 70 (6), 1068–1074. doi: 10.1093/cid/ciz319 31321410

[B23] Martin G. S. Mannino D. M. Moss M. (2006). The Effect of Age on the Development and Outcome of Adult Sepsis. Crit. Care Med. 34 (1), 15–21. doi: 10.1097/01.ccm.0000194535.82812.ba 16374151

[B24] McCann S. Byrne J. L. Rovira M. Shaw P. Ribaud P. Sica S.. (2004). Outbreaks of Infectious Diseases in Stem Cell Transplant Units: A Silent Cause of Death for Patients and Transplant Programmes. Bone Marrow. Transplant. 33 (5), 519–529. doi: 10.1038/sj.bmt.1704380 14743201

[B25] Mylotte J. M. Tayara A. (2000). Staphylococcus Aureus Bacteremia: Predictors of 30-Day Mortality in a Large Cohort. Clin. Infect. Dis. 31 (5), 1170–1174. doi: 10.1086/317421 11073748

[B26] Satlin M. J. Cohen N. Ma K. C. Gedrimaite Z. Soave R. Askin G.. (2016). Bacteremia Due to Carbapenem-Resistant Enterobacteriaceae in Neutropenic Patients With Hematologic Malignancies. J. Infect. 73 (4), 336–345. doi: 10.1016/j.jinf.2016.07.002 27404978 PMC5026910

[B27] Shipman P. Highland J. Witt B. Alt J. (2021). Non-Invasive Fungal Sinusitis as a Complication of a Steroid-Eluting Stent Following Endoscopic Sinus Surgery: A Case Report. Ann. Otol. Rhinol. Laryngol. 5, 34894211036844. doi: 10.1177/00034894211036844 34350789

[B28] Si X. Ji G. Ma S. Xu Y. Zhao J. Zhang Y.. (2021). In-Situ-Sprayed Dual-Functional Immunotherapeutic Gel for Colorectal Cancer Postsurgical Treatment. Adv. Healthc. Mater. 10 (20), e2100862. doi: 10.1002/adhm.202100862 34347370

[B29] Stoma I. Karpov I. Uss A. Krivenko S. Iskrov I. Milanovich N.. (2019). Combination of Sepsis Biomarkers May Indicate an Invasive Fungal Infection in Haematological Patients. Biomarkers 24 (4), 401–406. doi: 10.1080/1354750X.2019.1600023 30907674

[B30] Torres V. B. Azevedo L. C. Silva U. V. Caruso P. Torelly A. P. Silva E.. (2015). Sepsis-Associated Outcomes in Critically Ill Patients With Malignancies. Ann. Am. Thorac. Soc. 12 (8), 1185–1192. doi: 10.1513/AnnalsATS.201501-046OC 26086679

[B31] Turkkan S. Beyoglu M. A. Sahin M. F. Yazicioglu A. Tezer Tekce Y. Yekeler E. (2021). COVID-19 in Lung Transplant Recipients: A Single-Center Experience. Transpl. Infect. Dis. 23 (5), e13700. doi: 10.1111/tid.13700 34323353 PMC8420517

[B32] Willis M. L. Mahung C. Wallet S. M. Barnett A. Cairns B. A. Coleman L. G. Jr. (2021). Plasma Extracellular Vesicles Released After Severe Burn Injury Modulate Macrophage Phenotype and Function. J. Leukoc. Biol. 111 (1), 33–49. doi: 10.1002/JLB.3MIA0321-150RR 34342045 PMC8716518

[B33] Zduniak A. Mihailescu S. D. Lequesne J. Lenain P. Contentin N. Pepin L. F.. (2021). Outcomes After Intensive Care Unit Admission in Newly Diagnosed Diffuse Large B-Cell Lymphoma Patients: A Real-Life Study. Eur. J. Haematol. 106 (6), 788–799. doi: 10.1111/ejh.13606 33624346

[B34] Zhang J. Du Z. Bi J. Wu Z. Lv Y. Zhang X.. (2019). Comparison of Clinical Characteristics and Outcomes of Pyogenic Liver Abscess Patients < 65 Years of Age Versus ≥ 65 Years of Age. BMC Infect. Dis. 19 (1), 233. doi: 10.1186/s12879-019-3837-2 30845927 PMC6407260

