# Supplementary material for: Clinical Characteristics of Bloodstream Infection in Immunosuppressed Patients: A 5-Year Retrospective Cohort Study
Source: Front Cell Infect Microbiol. 2022 Apr 4;12:796656. doi: 10.3389/fcimb.2022.796656 (PMC9014008; doi:10.3389/fcimb.2022.796656)
Supplement: Supplementary file 1 [file Table_1.pdf]

Supplementary table 1 Clinical characteristics of bloodstream infection with 60-day survivals in immunosuppressive patients

N (Total=896)	60-day survivals (Total=698)	60-day death (Total=198)	*P* Value
**Characteristics**			
Age, mean (years)	58.15±16.83	62.45±17.10	0.002
BMI	22.94±3.77	23.54±3.97	0.051
PCT>0.5 ug/L	11.40±31.89	20.49±43.59	0.007
Sex (n, %)			0.370
Male	462(66.19%)	141(71.21%)	
Female	236(33.81%)	57(28.79%)	
Smoking	207(29.66%)	58(29.29%)	0.954
Drinking	152(27.78%)	39(19.70%)	0.549
Age>60 years (n, %)	358(51.29%)	127(64.14%)	0.001
ICU-Stay (n, %)	270(38.68%)	78(39.40%)	0.817
**Underlying diseases (n, %)**			
Hypertension	300(42.98%)	99(50%)	0.079
Diabetes	180(25.79%)	59(29.78%)	0.260
Asthma	5(0.72%)	4(2.02%)	0.115
COPD	21(3.01%)	4(2.02%)	0.626
Chronic Bronchitis	15(2.15%%)	5(2.52%)	0.752
Cerebral Infarction	48(6.88%)	14(7.07%)	0.924
Coronary Heart Disease	100(14.33%)	28(14.14%)	0.948
Anemia	72(10.32%)	23(11.62%)	0.600
Hypoproteinemia	68(9.89%)	19(9.60%)	0.951
Hyperlipidemia	26(3.72%)	10(5.05%)	0.402
Chronic renal failure	19(2.72%)	6(3.03%)	0.816
Pulmonary hypertension	28(4.01%)	4(2.02%)	0.276
