# Supplementary material for: Clinical Characteristics of Bloodstream Infection in Immunosuppressed Patients: A 5-Year Retrospective Cohort Study
Source: Front Cell Infect Microbiol. 2022 Apr 4;12:796656. doi: 10.3389/fcimb.2022.796656 (PMC9014008; doi:10.3389/fcimb.2022.796656)
Supplement: Supplementary file 2 [file Table_2.pdf]

Supplementary table 2 Primary illness characteristics of bloodstream infection with 60-day survivals in immunosuppressive patients

| N (Total=896) | 60-day survivals（Total=698） | 60-day death (Total=198) | *P* Value |
|---|---|---|---|
| **Underlying conditions (n, %)** | | | |
| Liver Cirrhosis | 27(3.89%) | 10(5.05%) | 0.461 |
| Acute myocardial infarction | 14(2.01%) | 9(4.54%) | 0.046 |
| Acute pancreatitis | 56(8.02%) | 13(6.60%) | 0.497 |
| Rheumatic heart disease | 36(5.18%) | 8(4.04%) | 0.521 |
| Infective endocarditis | 16(2.29%) | 7(3.53%) | 0.329 |
| Valvular heart disease | 16(2.29%) | 5(2.52%) | 0.848 |
| Rheumatic systemic diseases | 11(1.58%) | 2(1.01%) | 0.744 |
| Skin disease | 7(1.00%) | 5(2.52%) | 0.100 |
| Burning | 46(6.59%) | 11(5.56%) | 0.599 |
| Biliary infection | 40(5.73%) | 5(2.52%) | 0.068 |
| Aortic dissection | 19(2.72%) | 7(3.53%) | 0.547 |
| Aortic aneurysm | 13(1.82%) | 1(0.50%) | 0.326 |
| Tumors | 257(36.82%) | 81(40.91%) | 0.295 |
