# Supplementary material for: Clinical Characteristics of Bloodstream Infection in Immunosuppressed Patients: A 5-Year Retrospective Cohort Study
Source: Front Cell Infect Microbiol. 2022 Apr 4;12:796656. doi: 10.3389/fcimb.2022.796656 (PMC9014008; doi:10.3389/fcimb.2022.796656)
Supplement: Supplementary file 3 [file Table_3.pdf]

Supplementary table 3. Clinical characteristics of tumor classification with 60-day survivals

N (Total=338)	60-day survivals （Total=257）	60-day death (Total=81)	*P* Value
Haematological diseases	88(34.24%)	29(35.80%)	0.452
Gastrointestinal cancer	136(52.92%)	42(51.85%)	0.591
Respiratory system tumors	10(3.89%)	4(4.94%)	0.556
Peritoneal malignant tumor	11(4.28%)	3(3.70%)	1.000
Urinary system tumors	8(3.11%)	2(2.47%)	1.000
