# Supplementary material for: Clinical Characteristics of Bloodstream Infection in Immunosuppressed Patients: A 5-Year Retrospective Cohort Study
Source: Front Cell Infect Microbiol. 2022 Apr 4;12:796656. doi: 10.3389/fcimb.2022.796656 (PMC9014008; doi:10.3389/fcimb.2022.796656)
Supplement: Supplementary file 4 [file Table_4.pdf]

Supplementary table 4. Clinical characteristics of bloodstream infection with 60-day survivals in immunosuppressive patients

N (Total=896)	60-day survivals （Total=698）	60-day death (Total=198)	*P* Value
**Inducement (n, %)**			
Surgery	331(47.42%)	101(51.01%)	0.382
Chemotherapy	57(8.17%)	19(9.60%)	0.528
Burning	46(6.59%)	11(5.56%)	0.599
Catheter placement	20(2.87%)	4(2.02%)	0.625
Transplant status	24(3.43%)	5(2.53%)	0.522
Hormone	18(2.58%)	4(2.02%)	0.799
Hormone+ Immunosuppressant	14(2.01%)	5(2.53%)	0.654
**Complications (n, %)**			
Acute renal failure	92(13.18%)	22(11.11%)	0.441
Cerebral hemorrhage	11(1.58%)	4(2.02%)	0.667
Abdominal infection	64(9.17%)	18(9.09%)	0.973
Abdominal hemorrhage	16(2.29%)	5(2.53%)	0848
Acute respiratory failure	179(25.64%)	43(21.72%)	0.259
Gastrointestinal bleeding	29(4.15%)	9(4.55%)	0.810
Shock	174(24.93%)	40(20.20%)	0.619
**Pathogen Sources (n, %)**			
Respiratory tract	82(11.75%)	22(11.11%)	0.830
Digestive tract	193(27.65%)	64(32.32%)	0.213
Skin	66(9.46%)	17(8.59%)	0.709
Urinary system	14(2.01%)	5(2.53%)	0.654
Catheter	15(2.15%)	4(2.02%)	1.000
**Etiologies (n, %)**			
ABA	44(6.30%)	12(6.01%)	1.000
ECO	119(17.05%)	25(12.63%)	0.135
EFA	27(3.87%)	10(5.05%)	0.461
EFM	37(5.30%)	11(5.56%)	0.888
KPN	140(20.06%)	48(24.24%)	0.202
PAE	42(6.02%)	9(4.55%)	0.430
PMA	21(3.01%)	2(1.01%)	0.133
SAU	71(10.17%)	18(9.09%)	0.654
SCA	12(1.72%)	5(2.53%)	0.463
SEP	62(8.88%)	14(7.07%)	0.419
SHL	24(3.44%)	10(5.05%)	0.295
SHO	9(1.29%)	5(2.53%)	0.216
Others	90(12.89%)	28(14.14%)	Not Applicable
**Antibacterial therapy**			
Piperacillin-tazobactam	19(2.72%)	8(4.04%)	0.409
Cefoperazone-sulbactam	67(9.60%)	15(7.58%)	0.284
Carbapenems	424(60.74%)	142(71.72%)	0.056
Second generation cephalosporins	15(2.15%)	1(0.51%)	0.190
Third generation cephalosporins	134(19.20%)	30(15.15%)	0.110
Fourth generation cephalosporins	14(2.01%)	6(3.03%)	0.445
Daptomycin	10(1.43%)	3(1.52%)	1.000
Fluoroquinolones	92(13.18%)	19(9.60%)	0.113

Vancomycin	198(28.37%)	67(33.84%)	0.302
Linezolid	105(15.04%)	28(14.14%)	0.555
Macrolides	3(0.43%)	0(0)	Not Applicable
Trimethoprim-sulfamethoxazole (TMP-SMX)	5(0.72%)	3(1.52%)	0.580
