# Supplementary figures and images for: Clinical Characteristics of Bloodstream Infection in Immunosuppressed Patients: A 5-Year Retrospective Cohort Study
Source: Front Cell Infect Microbiol. 2022 Apr 4;12:796656. doi: 10.3389/fcimb.2022.796656 (PMC9014008; doi:10.3389/fcimb.2022.796656)

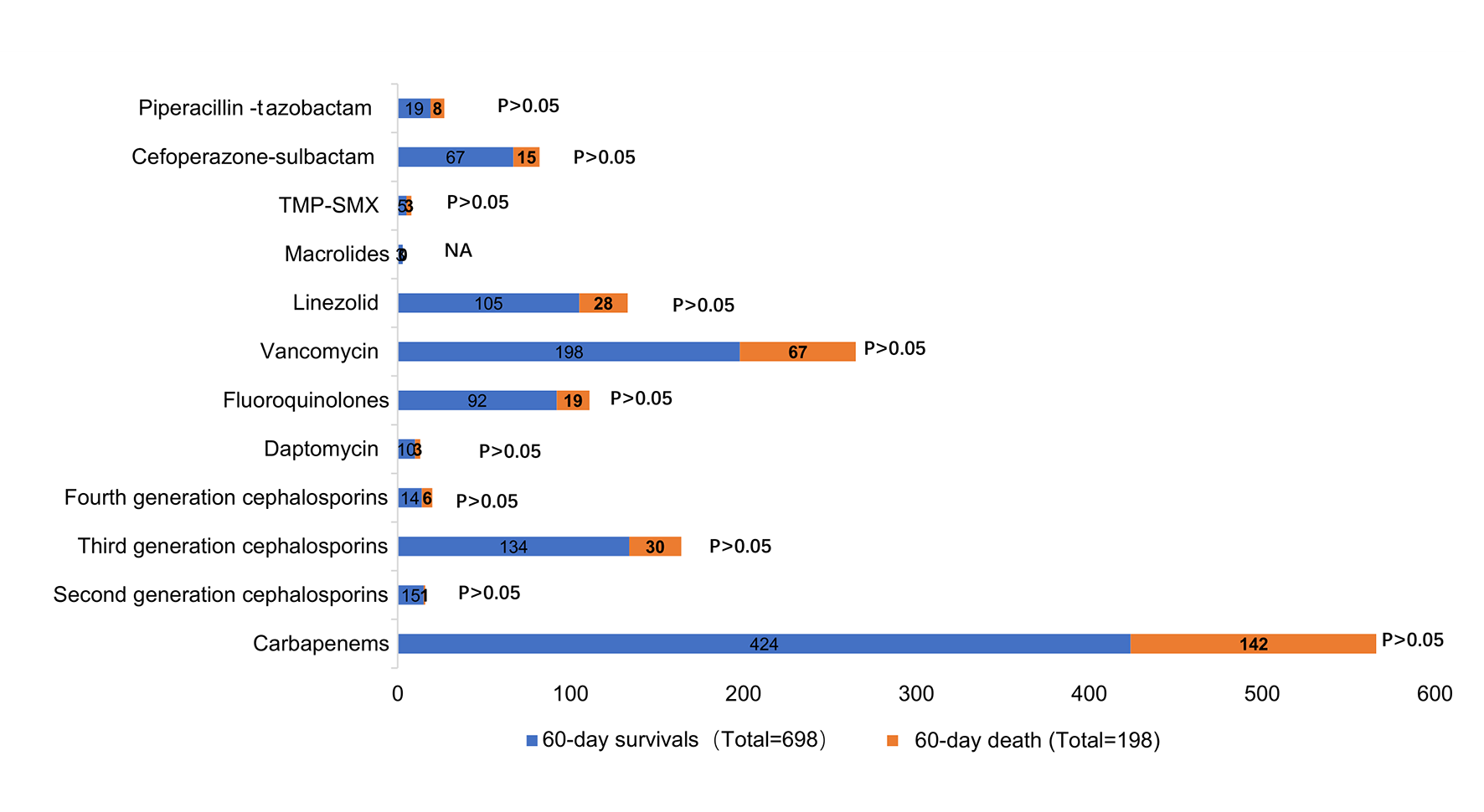

Supplement: Supplementary Figure 1 — Distribution of bloodstream infection according to 60-day survivals and 60-day death stratifications: different antibacterial therapy. [file Image_1.tif]

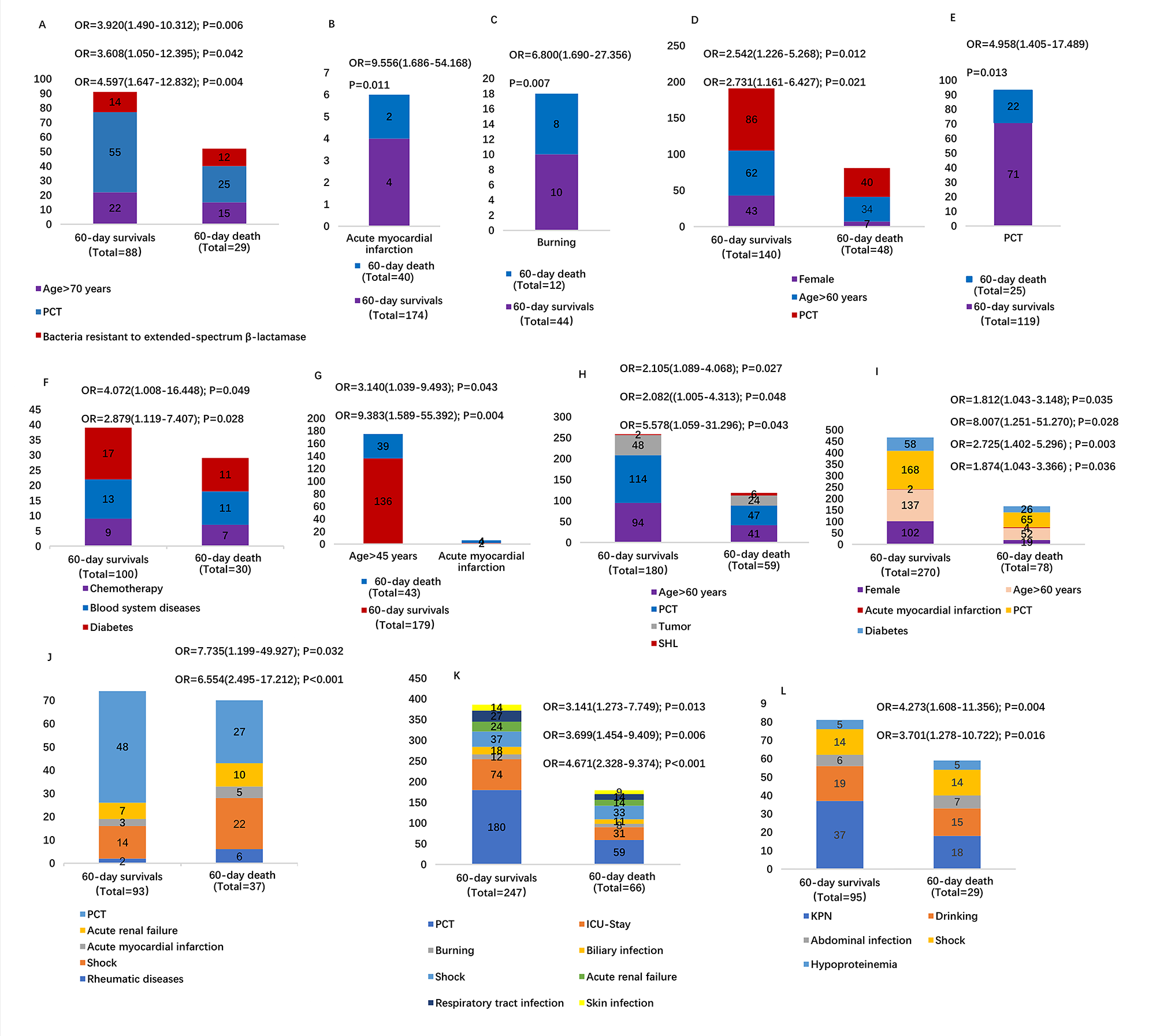

Supplement: Supplementary Figure 2 — Risk factors associated with 60-day mortality in different subgroups. [file Image_2.tif]
